# Increased calcium intake is associated lower serum 25-hydroxyvitamin D levels in subjects with adequate vitamin D intake: a population-based observational study

**DOI:** 10.1186/s40795-020-00381-4

**Published:** 2020-11-02

**Authors:** Rolf Jorde, Guri Grimnes

**Affiliations:** 1 Tromsø Endocrine Research Group, Department of Clinical Medicine, UiT The Arctic University of Norway, Tromsø, Norway; 2 Division of Internal Medicine, University Hospital of North Norway, 9038 Tromsø, Norway

**Keywords:** Calcium, Parathyroid hormone, Vitamin D, 25-hydroxyvitamin D

## Abstract

**Background:**

There are indications that an increased intake of calcium has a vitamin D sparing effect, which might be explained by a decreased catabolism of 25-hydroxyvitamin D (25(OH)D). However, there are only a few studies where this has been examined.

**Method:**

In the seventh survey of the Tromsø study, serum 25(OH)D and parathyroid hormone were measured, and questionnaires on calcium and vitamin D intakes filled in.

**Results:**

There were significant interactions between sex, calcium and vitamin D intakes regarding serum 25(OH)D level. The analyses were therefore done stratified. In males there was, regardless of vitamin D intake, a significant decrease in serum 25(OH)D with increasing calcium intake. The difference in serum 25(OH)D between those with the highest and lowest calcium intakes was approximately 10%. In the females, there was in subjects with low vitamin D intake (< 7 μg/d) a significant increase in serum 25(OH)D with increasing calcium intake, which could not be explained by secondary hyperparathyroidism. In females with higher vitamin D intakes, increasing calcium intake was associated with lower serum 25(OH)D levels.

**Conclusions:**

There is, at least in subjects with an adequate vitamin D intake, a negative association between calcium intake and serum 25(OH)D.

## Background

Vitamin D is obtained from the diet or supplements and also from production in the skin upon sun exposure. Vitamin D is first hydroxylated in the liver to 25-hydroxyvitamin D (25(OH)D), and then in the kidneys to 1,25-dihydroxyvitamin D (1,25(OH)_2_D) which is the active form of the vitamin. The main biological effect of 1,25(OH)_2_D is to increase the intestinal calcium absorption. The 25-hydroxylation is substrate driven, whereas the 1-hydroxylation is tightly regulated by parathyroid hormone (PTH). If the serum calcium level falls, the serum PTH level increase stimulating secretion of 1,25(OH)_2_D and thereby restoration of the serum calcium level [1].

The serum level of 25(OH)D is generally considered as the best marker of a subjects vitamin D status, due to its ease of measurement, long half-life and correlation with known vitamin D effects. However, there is no consensus as to what can be considered an adequate or optimal serum 25(OH)D level [2, 3].

Vitamin D deficiency may in children lead to rickets and in adults to osteomalacia. At least for the prevention of rickets, it appears that a high calcium intake can compensate for a low vitamin D intake and vice versa [4]. There are also indications that there might be an interaction between these intakes, so that a high calcium intake cause an increase in the serum 25(OH)D level as reviewed by Heaney [5]. Thus, rats fed a high calcium diet had a higher serum 25(OH)D level than rats on a low calcium diet [6], and in rats the inactivation of 25(OH)D in the liver is increased by calcium deprivation [7]. Furthermore, in subjects with primary or secondary hyperparathyroidism Davies et al. have shown that the half-life of 25(OH)D was lower in those with high 1,25(OH)_2_D levels. This could be explained by 1,25(OH)_2_D inducing the 24-hydroxylase which is the enzyme starting the degradation process of 25(OH)D as well as 1,25(OH)_2_D [8]. Based on these observations the concept of a vitamin D sparing effect of a high calcium intake has been suggested [9].

However, observational studies on calcium and vitamin D intakes regarding serum 25(OH)D levels have been equivocal. Thus, Olmos et al. found the serum 25(OH)D level to be positively correlated with dairy intake in 1811 individuals [10], as did Kinyamu in 243 women [11]. On the other hand, this was not confirmed by Hill et al. in a group of 1015 adolescents from Northern Ireland [12] nor by Andersen et al. in a group of 247 Pakistani living in Denmark and with low serum 25(OH)D levels [13]. Similarly, randomized controlled trials (RCTs) have shown a large and positive effect by calcium supplementation on the 25(OH)D response to vitamin D supplementation in one study [14], which has not been confirmed in others [15–18].

In the last Tromsø study serum 25(OH)D was measured and questionnaires on intakes of vitamin D and calcium filled in by more than 11,000 subjects. In a sub-group of 1917 subjects serum PTH was measured as well. This gave us the opportunity to evaluated interrelationships between these intakes regarding serum 25(OH)D level, and to evaluate if these relationships could be explained by secondary hyperparathyroidism, which to our knowledge has not been done in a huge cohort before.

## Methods

### Study design and participants

The Tromsøs study is a population-based health survey performed in Tromsø, Northern Norway at 69° North. The first survey was performed in 1974 and the seventh survey in 2015/2016. Originally the study focused on cardiovascular diseases and their prevention, but later surveys have included a wide range of tests and analyses [19]. In the seventh survey all citizens older than 40 years and living in the municipality of Tromsø were invited, and 64.7% (21,083) attended. The subjects filled in a general health questionnaire including intakes of vitamin D and calcium supplements, sunny vacation(s) last two months, and use of solarium. Intakes of vitamin D and calcium were estimated based on a validated food frequency questionnaire that included 261 questions on food items, meals, and beverages [20, 21]. Non-fasting blood samples were drawn; height and weight were measured wearing light clothing, and body mass index (BMI) calculated as kg/m^2^.

### Biochemical analyses

All biochemical analyses were performed at the Department of Medical Biochemistry, University Hospital of North Norway. Serum 25(OH)D was measured in all subjects and determined by an in-house-developed LC/MS-MS method as previously described in detail [22]. The laboratory takes part in the external quality program DEQAS. Serum PTH and calcium were measured in a subgroup of 3769 subjects who also had attended the fourth survey in 1994/1995. Serum PTH was analysed with an electrochemiluminescence immunoassay using an automated clinical chemistry analyser (Cobas 6000, Roche). Serum calcium was measured by an automated analyser (Modular P, Roche Diagnostics, Mannheim, Germany) with reagents from Boehringer Mannheim. Reference ranges for serum PTH were 1.1–6.8 pmol/L for those < 51 years and 1.1–7.5 pmol/L for those > 50 years, and for serum calcium 2.20–2.50 mmol/L.

### Statistical analyses

Normal distribution was evaluated by visual inspection of histograms and by calculation of skewness and curtosis, and considered normal for all dependent variables except for serum PTH. Serum PTH was therefore normalized by log transformation before use as dependent variable. Comparisons between groups were performed with Student’s t-test. For evaluation of predictors of serum 25(OH)D a linear regression model was used with covariates as indicated in the table. Interactions between calcium and vitamin D intake groups, gender and age regarding the serum 25(OH)D level were evaluated with a general linear model. Linear trends were evaluated with linear regression with adjustments for sex, age, BMI and month of blood sampling (with the use of dummy variables) as indicated in the tables.

All data presented in this study, including the biochemical analyses, are from the seventh survey of the Tromsø study. The data are shown as mean (95% CI). All tests were done two-sided, and a *P*-value < 0.05 was considered statically significant. The Bonferroni correction for multiple comparisons was used as indicated in the tables.

## Results

### Main study

A total of 13,743 subjects had completed datasets regarding vitamin D and calcium intakes and valid serum 25(OH)D measurement. Among these, 2466 had been on a recent sunny holiday and among the remainder, 27 were using solarium on a regular basis, leaving 11,250 subjects (5982 females and 5268 males) who were included in the present analyses. Among these, 6.7% used calcium tablets daily, and 37.2% used cod liver oil and/or omega-3 fish oil capsules and/or other supplements with vitamin D on a daily basis, and 12.7% were daily smokers. The mean serum 25(OH)D level was 62.2 nmol/L, and 29.6% of the subjects had serum 25(OH)D < 50 nmol/L. Other characteristics are shown in Table 1.

**Table 1:** Characteristics of the participants in the main cohort and the sub-study

	All subjects	Males	Females
Main cohort^a^			
N	11,250	5268	5982
Age (years)	58.3 (58.1, 58.5)	58.5 (58.2, 58.8)	58.0 (57.7, 58.3)*
BMI (kg/m^2^)	27.3 (27.2, 27.4)	27.7 (27.6, 27.8)	26.9 (26.8, 27.1)***
Serum 25(OH)D (nmol/L)	62.2 (62.0, 62.7)	59.2 (58.7, 59.8)	65.1 (64.5, 65.6)***
Vitamin D intake (ug/d)^c^	14.1 (14.0, 14.3)	14.5 (14.2, 14.7)	13.9 (13.6, 14.1)**
Calcium intake (mg/d)^c^	1188 (1177, 1199)	1247 (1231, 1264)	1136 (1121, 1151)***
Sub-study^b^			
N	1917	828	1089
Age (years)	70.2 (69.8, 70.6)	70.7 (70.3, 71.3)	69.9 (69.3, 70.4)
BMI (kg/m^2^)	27.4 (27.2, 27.6)	27.6 (27.3, 27.8)	27.4 (27.1, 27.6)
Serum 25(OH)D (nmol/L)	68.2 (67.3, 69.2)	65.3 (64.0, 66.7)	70.4 (69.2, 71.7)***
Serum PTH (pmol/L)	5.8 (5.7, 5.9)	5.9 (5.7, 6.0)	5.8 (5.7, 5.9)
Serum calcium (mmol/L)	2.36 (2.36, 2.36)	2.35 (2.35, 2.36)	2.37 (2.36, 2.37)***
Vitamin D intake (ug/d)^c^	14.9 (14.4, 15.4)	15.2 (14.5, 15.9)	14.6 (14.0, 15.3)
Calcium intake (mg/d)^c^	1168 (1140, 1195)	1199 (1157, 1241)	1144 (1108, 1181)

*P < 0.05, **P < 0.01, ***P < 0.001. Student’s t-test versus males

^a^ Subjects in the Tromsø study with valid food frequency questionnaires, serum 25(OH) D measurement, not been on a sunny vacation last 2 months, and not taking solarium regularly

^b^ As in the main cohort but with valid serum PTH measurement and with serum calcium < 2.51 mmol/L

^c^Including supplements

The data are mean (95% CI). The *P* values are not adjusted for multiple testing

In a linear regression model with serum 25(OH)D as dependent variable, female gender, age and vitamin D intake were significant positive predictors of serum 25(OH)D, whereas BMI and calcium intake were significant negative predictors (Supplemental Table 1).

However, there was a significant interaction between calcium and vitamin D intakes regarding 25(OH)D (*P* < 0.001). Furthermore, when doing this analysis stratified for vitamin D intake, there was in the lowest vitamin D intake group (< 7 μg/d) a significant interaction between sex and calcium intake regarding serum 25(OH)D (*P* < 0.01). The relations between serum 25(OH)D, vitamin D and calcium intakes were therefore analysed for all subjects together as well as sex stratified. There were no interactions with age.

**Fig. 1:**
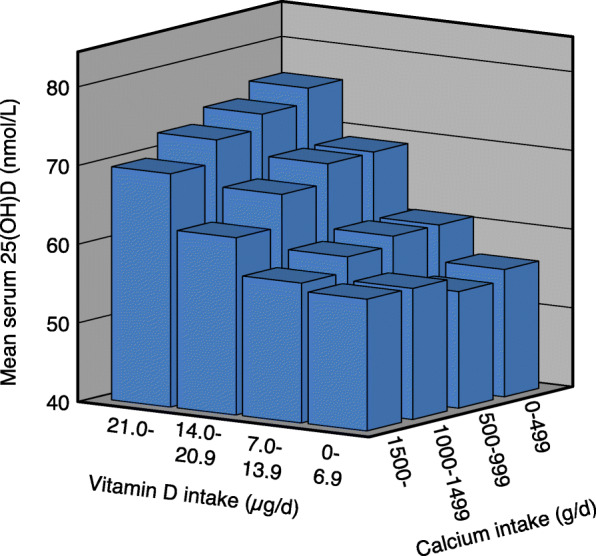
Serum 25(OH)D in relation to vitamin D and calcium intakes in 11,250 subjects who have not been on a sunny vacation last two months, and not taking solarium regularly

**Fig. 2:**
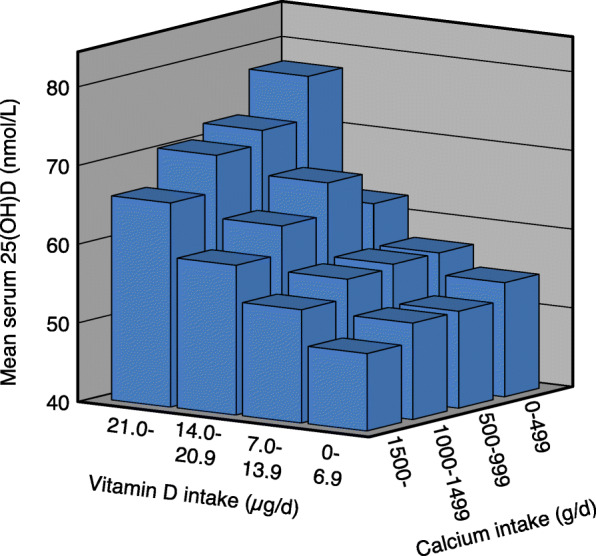
Serum 25(OH)D in relation to vitamin D and calcium intakes in 5268 males who have not been on a sunny vacation last two months, and not taking solarium regularly

**Fig. 3:**
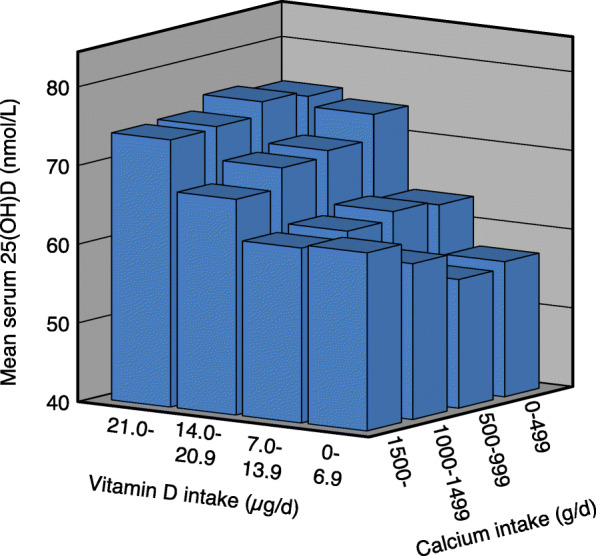
Serum 25(OH)D in relation to vitamin D and calcium intakes in 5982 females who have not been on a sunny vacation last two months, and not taking solarium regularly

With increasing vitamin D intake there was a significant increase in serum 25(OH)D in all calcium intake groups in both genders. With increasing calcium intake there was in the low vitamin D intake group (< 7 μg/d) a significant increase in serum 25(OH)D in the females but not in the males. In the males and when both genders were analysed together, there was with increasing calcium intake a non-significant decrease in serum 25(OH)D in the 7–13.9 μg vitamin D/d intake group, whereas in the two highest vitamin D intake groups (14.0–20.9 and > 20.9 μg/d) there was a significant decrease in serum 25(OH)D. In these two vitamin D intake groups the difference in serum 25(OH)D between those with the lowest and highest calcium intake was ~ 10% (Table 2 and Figs. 1, 2 and 3).

**Table 2:** Serum 25(OH)D in relation to calcium and vitamin D intakes in all subjects and gender specific in the 11,250 subjects in the main cohort

	Calcium intake§ < 500 mg/d		Calcium intake§ 500–999 mg/d		Calcium intake§ 1000–1499 mg/d		Calcium intake§ > 1499 mg/d	
	n	Serum 25(OH)D (nmol/L)	n	Serum 25(OH)D (nmol/L)	n	Serum 25(OH)D (nmol/L)	n	Serum 25(OH)D (nmol/L)
All subjects‡								
Vitamin D intake (ug/d)§								
< 7.0	444	56.2 (54.3, 58.0)	1396	54.8 (53.8, 56.7)	874	56.7 (55.2, 58.1)	302	56.7 (54.3, 59.1)
7.0–13.9	223	60.8 (58.1, 63.5)	1360	60.8 (59.8, 61.9)	1531	59.7 (58.7, 60.6)	943	57.8 (56.5, 59.0)#
14.0–20.9	69	69.1 (64.0, 74.1)	513	69.0 (67.3, 70.8)	624	66.6 (65.0, 68.2)	553	62.5 (60.8, 64.2)#**
> 20.9	77	76.2 (71.1, 81.2)†	666	74.3 (72.7, 76.0)†	871	72.5 (71.2, 73.8)†	804	69.6 (68.2, 71.1)#**†
Males‡								
Vitamin D intake (ug/d)§								
< 7.0	165	54.5 (51.7, 57.3)	530	52.3 (50.7, 53.9)	363	52.3 (50.3, 54.2)	139	49.8 (47.1, 52.6)
7.0–13.9	93	57.3 (53.0, 61.6)	635	57.3 (55.8, 58.8)	815	56.8 (55.6, 58.1)	536	54.4 (52.8, 56.0)
14.0–20.9	29	62.5 (57.4, 67.7)	208	66.6 (63.7, 69.5)	284	62.6 (60.5, 64.7)	321	59.0 (56.9, 61.1)#**
> 20.9	32	77.7 (68.9, 86.5)†	286	72.3 (69.9, 74.6)†	401	70.5 (68.7, 72.4)†	431	65.9 (64.1, 67.7)#**†
Females‡								
Vitamin D intake (ug/d)§								
< 7.0	279	57.2 (54.7, 59.7)	866	56.4 (55.0, 57.7)	511	59.8 (57.8, 61.8)	163	62.6 (59.1, 66.2)#*
7.0–13.9	130	63.4 (59.9, 66.8)	725	64.0 (62.5, 65.5)	716	62.9 (61.5, 64.4)	407	62.2 (60.4, 64.1)
14.0–20.9	40	73.9 (66.2, 81.6)	305	70.7 (68.5, 73.0)	340	70.0 (67.6, 72.3)	232	67.4 (64.6, 70.2)
> 20.9	45	75.1 (68.9, 81.4)†	380	75.9 (73.7, 78.1)†	470	74.2 (72.4, 76.0)†	373	73.9 (71.8, 76.1)†

#*P* < 0.01. Linear trend for serum 25(OH)D across calcium intake groups without adjustments

*P < 0.05, **P < 0.01. Linear trend for serum 25(OH)D across calcium intake groups with sex, age, BMI and month of blood sampling (using dummy variables) as covariates

†*P* < 0.001. Linear trend for serum 25(OH)D across vitamin D intake groups without adjustments as well as with sex, age, BMI and month of blood sampling (using dummy variables) as covariates

‡ Subjects in the Tromsø study with valid food frequency questionnaires, serum 25(OH)D measurement, not been on a sunny vacation last 2 months, and not taking solarium regularly

§Including supplements

The data are mean (95% CI). The *P* values are adjusted for multiple testing with a factor of 4

### Sub-group study

A total of 1917 subjects (1089 females and 828 males) had valid data-sets as above, valid serum PTH measurement and serum calcium < 2.51 nmol/L, and were included in the sub-group analyses. Among these, 11.8% used calcium tablets daily, and 53.2% used cod liver oil and/or omega-3 fish oil capsules and/or other supplements with vitamin D on a daily basis. Their other baseline characteristics are shown in Table 1. In general, these subjects were older and had higher serum 25(OH)D levels than those in the main cohort.

**Fig. 4:**
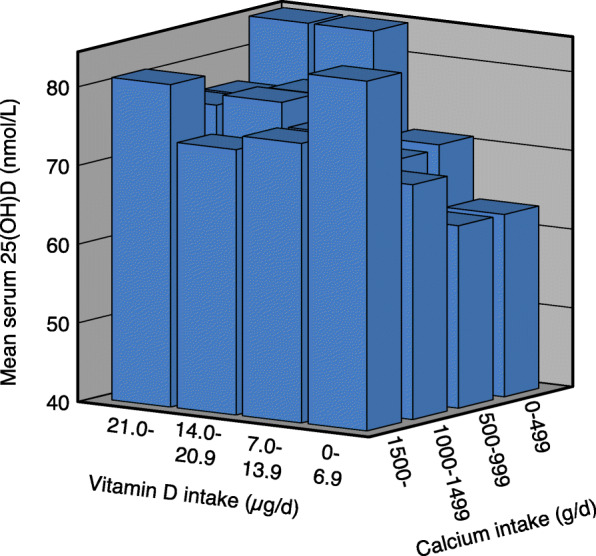
Serum 25(OH)D in relation to vitamin D and calcium intakes in 522 females with serum PTH < 5.35 pmol/L, who have not been on a sunny vacation last two months, and not taking solarium regularly

**Fig. 5:**
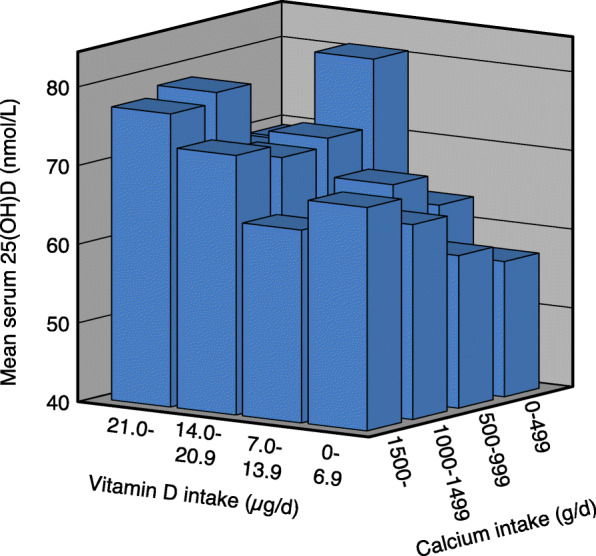
Serum 25(OH)D in relation to vitamin D and calcium intakes in 567 females with serum PTH > 5.35 pmol/L, who have not been on a sunny vacation last two months, and not taking solarium regularly

As expected the serum PTH levels were highest in the subjects with the lowest calcium and vitamin D intakes. However, this was only significant for a few of the vitamin D and calcium intake subgroups (Table 3). As in the main study there was in the females a significant (*P* < 0.001) increase in serum 25(OH)D with increasing calcium intake in those with a low vitamin D intake, whereas there in both genders was a decrease in serum 25(OH)D with increasing calcium intake in those with higher vitamin D intakes (Table 4). The same pattern was seen if dividing the cohort in subjects with serum PTH below or above 5.35 pmol/L (the median PTH level), which is illustrated for the females in Figs. 4 and 5.

**Table 3:** Serum PTH in relation to calcium and vitamin D intakes in all subjects and gender specific in 1917 subjects in the sub-study

	Calcium intake§ < 500 mg/d		Calcium intake§ 500–999 mg/d		Calcium intake§ 1000–1499 mg/d		Calcium intake§ > 1499 mg/d	
	n	Serum PTH (pmol/L)	n	Serum PTH (pmol/L)	n	Serum PTH (pmol/L)	n	Serum PTH (pmol/L)
All subjects‡								
Vitamin D intake (ug/d)§								
< 7.0	86	6.3 (5.8, 6.9)	230	5.9 (5.6, 6.2)	125	5.8 (5.5, 6.2)	53	5.7 (5.0, 6.5)
7.0–13.9	44	6.6 (5.7, 7.4)	225	5.9 (5.7, 6.2)	236	5.9 (5.6, 6.1)	119	6.0 (5.6, 6.4)
14.0–20.9	15	7.4 (5.0, 9.8)	85	5.8 (5.4, 6.3)	130	5.6 (5.3, 6.0)	104	5.8 (5.3, 6.2)
> 20.9	16	5.7 (4.5, 6.9)	143	5.7 (5.4, 6.1)	151	5.4 (5.0, 5.9)	155	5.3 (5.1, 5.6)
Males‡								
Vitamin D intake (ug/d)§								
< 7.0	28	6.9 (5.6, 8.2)	92	5.8 (5.4, 6.2)	44	5.8 (5.2, 6.5)	19	5.9 (4.4, 7.5)
7.0–13.9	21	7.0 (5.4, 8.6)	103	6.1 (5.6, 6.6)	107	6.0 (5.6, 6.4)	66	6.1 (5.6, 6.7)
14.0–20.9	4	5.7 (4.4, 6.9)	33	5.6 (4.9, 6.4)	56	5.6 (5.0, 6.2)	50	6.1 (5.4, 6.8)
> 20.9	4	5.7 (1.2, 9.5)	68	5.6 (5.2, 6.0)	62	5.6 (4.5, 6.7)	71	5.2 (4.9, 5.6)†
Females‡								
Vitamin D intake (ug/d)§								
< 7.0	58	6.1 (5.6, 6.6)	138	6.0 (5.6, 6.4)	81	5.8 (5.4, 6.2)	34	5.6 (4.7, 6.5)
7.0–13.9	23	6.1 (5.3, 6.9)	122	5.8 (5.5, 6.1)	129	5.8 (5.4, 6,2)	53	5.8 (5.2, 6.4)
14.0–20.9	11	8.0 (4.7, 11.4)	52	6.0 (5.3, 6.6)	74	5.7 (5.3, 6.1)	54	5.4 (5.0, 5.9)#
> 20.9	12	5.7 (4.2, 7.2)	75	5.8 (5.3, 6.3)	89	5.3 (5.0, 5.7)	84	5.4 (5.0, 5.8)

#*P* < 0.05. Linear trend for serum PTH across calcium intake groups without adjustments

†*P* < 0.05. Linear trend for serum PTH across vitamin D intake groups without adjustments as well as with sex, age, BMI and month of blood sampling (using dummy variables) as covariates

‡ Subjects in the Tromsø study with valid food frequency questionnaires, serum 25(OH)D measurement, not been on a sunny vacation last 2 months, not taking solarium regularly, with valid serum PTH measurement, and with serum calcium < 2.51 mmol/L.

§ Including supplements

The data are mean (95% CI). The P values are adjusted for multiple testing with a factor of 4

**Table 4:** Serum 25(OH)D in relation to calcium and vitamin D intakes in all subjects and gender specific in 1917 subjects in the sub-study

	Calcium intake§ < 500 mg/d		Calcium intake§ 500–999 mg/d		Calcium intake§ 1000–1499 mg/d		Calcium intake§ > 1499 mg/d	
	n	Serum 25(OH)D (nmol/L)	n	Serum 25(OH)D (nmol/L)	n	Serum 25(OH)D (nmol/L)	n	Serum 25(OH)D (nmol/L)
All subjects‡								
Vitamin D intake (ug/d)§								
< 7.0	86	59.4 (55.4, 63.5)	230	60.4 (57.9, 62.9)	125	63.8 (60.2, 67.4)	53	69.3 (62.2, 76.4)##*
7.0–13.9	44	61.7 (55.3, 68.1)	225	66.2 (63.4, 69.1)	236	66.2 (63.6, 68.8)	119	65.3 (62.0, 68.6)
14.0–20.9	15	81.6 (70.3, 92.8)	85	75.1 (69.9, 80.2)	130	71.2 (67.7, 74.7)	104	67.8 (64.1, 71.4)#*
> 20.9	16	81.1 (68.0, 94.2)†	143	74.5 (71.3, 77.6)†	151	76.6 (73.5, 79.8)†	155	75.4 (72.4, 78.5)†
Males‡								
Vitamin D intake (ug/d)§								
< 7.0	28	58.7 (51.2, 65.5)	92	59.4 (55.7, 63.1)	44	57.8 (53.1, 62.6)	19	54.6 (45.9, 63.2)
7.0–13.9	21	56.6 (46.6, 66.6)	103	63.3 (59.4, 67.1)	107	63.8 (60.0, 67.6)	66	61.8 (57.4, 66.2)
14.0–20.9	4	76.6 (50.1, 103.0)	33	74.4 (64.2, 84.6)	56	67.1 (62.4, 71.8)	50	61.6 (56.3, 66.9)#*
> 20.9	4	81.8 (35.2, 128.5)	68	75.7 (70.6, 80.8)†	62	75.2 (70.9, 79.4)†	71	71.0 (67.3, 74.7)†
Females‡								
Vitamin D intake (ug/d)§								
< 7.0	58	59.8 (54.6, 64.9)	138	61.1 (57.8, 64.4)	81	67.1 (62.2, 71.9)	34	77.5 (68.5, 86.6)##**
7.0–13.9	23	66.3 (58.0, 74.7)	122	68.7 (64.6, 72.8)	129	68.2 (64.7, 71.7)	53	69.7 (64.7, 74.7)
14.0–20.9	11	83.4 (68.7, 98.1)	52	75.5 (69.7, 81.2)	74	74.3 (69.3, 79.3)	54	73.4 (68.7, 78.2)
> 20.9	12	80.9 (65.4, 96.3)†	75	73.3 (69.4, 77.3)†	89	77.7 (73.2, 82.2)†	84	79.2 (74.6, 83.7)

#*P* < 0.05, ## < 0.01. Linear trend for serum 25(OH)D across calcium D intake groups without adjustments

*P < 0.05, **P < 0.01. Linear trend for serum 25(OH)D across calcium intake groups with sex, age, BMI and month of blood sampling (using dummy variables) as covariates

†*P* < 0.001. Linear trend for serum 25(OH)D across vitamin D intake groups without adjustments as well as with sex, age, BMI and month of blood sampling (using dummy variables) as covariates

‡ Subjects in the Tromsø study with valid food frequency questionnaires, serum 25(OH)D measurement, not been on a sunny vacation last 2 months, not taking solarium regularly, with valid serum PTH measurement, and with serum calcium < 2.51 mmol/L

§ Including supplements

The data are mean (95% CI). The P values are adjusted for multiple testing with a factor of 4

## Discussion

In the present study we have found a negative association between calcium intake and serum 25(OH)D, except in females with a low vitamin D intake where the association was positive. Furthermore, these associations were seen in subjects with low as well as high serum PTH levels.

Our results are at difference with previous observational studies on calcium intake and serum 25(OH)D [14–18]. There are two likely explanations for this. Firstly, there was an interaction between calcium and vitamin D intakes as well as between genders regarding serum 25(OH)D, and analysing the cohorts without proper stratification as in our study could possibly mask the true relations. Furthermore, the difference in serum 25(OH)D level between those with high and low calcium intakes was approximately 10%, and accordingly, a large number of subjects need to be included, as in our study, to show significant effects.

It is difficult to find a plausible explanation for the mainly negative association between calcium intake and serum 25(OH)D. One could hypothesize that in a situation with a low calcium intake it would be important for the body to conserve its 25(OH)D stores and therefore reduce the 24-hydroxylation of 25(OH)D to the inactive 24,25(OH)_2_D form. However, low calcium intake and thereby low serum calcium levels stimulate the PTH secretion which increases the renal 1-hydroxylation of 25(OH)D to the active form 1,25(OH)_2_D which in turn increases the intestinal calcium absorption. This process cause removal of 25(OH)D from the circulation. Additionally, 1,25(OH)_2_D activates the 24-hydroxylase which starts the degradation and thus the elimination of 25(OH)D [1]. One would therefore expect that a low calcium intake should reduce, and not increase the serum 25(OH)D level. These PTH related mechanisms could therefore theoretically offer an explanation for the positive association between calcium intake and serum 25(OH)D in the females with low vitamin D intake. However, since the same patters of relation between calcium intake and serum 25(OH)D were seen when stratifying these females according to serum PTH levels, this cannot be the explanation.

The main calcium source is dairy products and subjects with a high calcium intake therefore also have a high phosphate intake, which will trigger the secretion of fibroblast growth factor 23 (FGF23). FGF23 will reduce the intestinal phosphate absorption by reducing the 1,25(OH)_2_D level through increased catabolism of 1,25(OH)_2_D as well as 25(OH)D [23]. Unfortunately we did not measure serum FGF23 nor serum phosphate and this explanation therefore purely speculative. Another explanation could be that the absorption of vitamin D was reduced by a high calcium intake which thereby would be associated with a lower serum 25(OH)D level. The absorption of vitamin D is assumed to be through passive diffusion as well as by membrane carriers, and may be increased by concomitant fat ingestion [24]. Furthermore, intestinal calcium ingestion may, by the formation of calcium-fatty acid soaps, which are excreted in the faeces, cause a slight reduction in intestinal fat absorption [25]. However, this reduction is less than 5 g/d [26], which is probably too low to significantly affect the vitamin D absorption and can therefore hardly explain our findings. Additionally, we have no plausible hypotheses for why the positive association between calcium intake and serum 25(OH)D in subjects with low vitamin D intake was seen in women only, since there was no major gender differences regarding calcium and vitamin D intakes, age, BMI and PTH. If our findings are not the results of unaccounted for confounding, we therefore have to postulate the existence of unknown physiological process that regulate the vitamin D metabolism in response to calcium intake. In this regard the concept of the personal vitamin D response index should be considered [27]. According to this concept, the molecular response to supplementation with vitamin D, such as changes in the epigenetic status and the respective transcription of genes, varies considerably between individuals. Hence, the measured serum 25(OH)D may not necessarily reflect the true impact of vitamin D and calcium supplementation on vitamin D metabolism in a given individual.

Our study was purely observational and we can therefore not imply causality between calcium intake and the serum 25(OH)D level. This has to be tested in randomized controlled trials (RCTs) for which there are only a few relevant ones. Thus, in a study by Goussous et al., 52 subjects were given 800 IU vitamin D/d and randomized to 500 mg calcium twice daily or placebo for 90 days. In the calcium group the mean serum 25(OH)D increased 16.2 nmol/L versus 17.4 nmol/L in the control group [16]. Similarly, in a study by McCullough et al. 92 subjects were randomized to 800 IU vitamin D/d, 2000 mg calcium, both or placebo for 6 months. The increase in serum 25(OH)D was almost identical, 25 and 26 nmol/L respectively, in those with and without additional calcium [17]. In a study by Grant et al. on prevention of low trauma fractures, 5292 subjects were randomized to 800 IU vitamin D/d, 1000 mg calcium/d, both or placebo, and serum 25(OH)D measured before and after one year in a subgroup of 60 subjects. In those allocated to combined vitamin D and calcium, serum 25(OH)D rose by 24.0 nmol/L, whereas the increase in those given vitamin D alone was 24.3 nmol/L [18]. And finally, in a study by Cashman et al. including 125 subjects stratified according to calcium intake and given 800 IU vitamin D/d, there was no evidence of a 25(OH)D sparing effect of high calcium intake. However, in the subjects with a low calcium intake (< 700 mg/d) the increase in mean serum 25(OH)D was non-significantly higher than in those with a high calcium intake (> 1000 mg/d) (26.1 nmol/L vs 20.0 nmol/l) [15].

All of these studies have included relatively few subjects. Given that the effect probably is small and may be different in subgroups, larger studies are needed to settle this question. It is unlikely that a large RCT will be designed specifically for this purpose, but there is at least one large study performed with vitamin D intervention with or without additional calcium and with serum 25(OH)D measurements. Thus, in the study by Baron et al. 2259 participants with recently diagnosed adenomas and no known colorectal polyps remaining after colonoscopy, were assigned to vitamin D 1000 IU/d, calcium 1200 mg/d, both or neither, with recurrence of adenoma as primary endpoint. Serum 25(OH)D was measured at baseline and after 1 year. However, the serum 25(OH)D increase in the vitamin D alone vs the vitamin D plus calcium groups was not (as far as we have been able to find) specifically reported in their publications [28].

Our study has several limitations. Firstly, as an observational study we cannot conclude about causality. We did not measure serum 1,25(OH)_2_D and 24,25(OH)_2_D, nor did we do gene-expression studies, which would have been important from a mechanistically point of view. Our subjects had a mean age of 58 years and only 29.6% were vitamin D deficient (serum 25(OH)D < 50 nmol/L). Our results are therefore not relevant for children at risk of rickets. Although our study was population based, the subjects in the sub-study were highly selected with mean age ~ 70 years and with mean serum 25(OH)D as high as 70 nmol/L. On the other hand, our study also has strengths as we included a large number of subjects, which made it meaningful to do stratified analyses. The observation of a negative association between calcium intake and serum 25(OH)D, at least in subjects with adequate vitamin D intake, was statistically highly significant and might reflect unknown aspects of vitamin D metabolism. Direct or indirect inhibition of 25-hydroxylation and/or stimulation of 25(OH)D degradation by calcium are the most obvious pathways, that potentially could be verified by gene-expression studies.

## Conclusions

We have found serum 25(OH)D to decrease with increasing calcium intake, at least in subjects with an adequate intake of vitamin D. Our findings are hard to explain, but if confirmed in large intervention trials, studies aiming at explaining the underling mechanisms should be encouraged.

## Supplementary information

**Additional file 1: Table S1.** A linear regression model with serum 25(OH)D as dependent variable and sex, age, BMI vitamin D and calcium intakes as covariates.

## Data Availability

The datasets used in the study are not available publicly but can be made available upon application to the Tromsø Study (tromsous@uit.no) (www.tromsoundersokelsen.no).

## Abbreviations

BMI: Body mass index
FGF23: Fibroblast growth factor 23
PTH: Parathyroid hormone
RCTs: Randomized controlled trials
1,25(OH)_2_D: 1,25-dihydroxyvitamin D
25(OH)D: 25-hydroxyvitamin D
