# Supplementary material for: Increased calcium intake is associated lower serum 25-hydroxyvitamin D levels in subjects with adequate vitamin D intake: a population-based observational study
Source: BMC Nutr. 2020 Nov 2;6:49. doi: 10.1186/s40795-020-00381-4 (PMC7604942; doi:10.1186/s40795-020-00381-4)
Supplement: Supplementary file 1 — Additional file 1: Table S1. A linear regression model with serum 25(OH)D as dependent variable and sex, age, BMI vitamin D and calcium intakes as covariates. [file 40795_2020_381_MOESM1_ESM.docx]

**Supplementary Table 1**. Linear regression models including 11,250 subjects participating in the Tromsø study with serum 25(OH)D as dependent variable

| Covariates |  | Standardized beta coefficients  __________________________________________________________________________________ | | | | |
| --- | --- | --- | --- | --- | --- | --- |
|  |  | Model 1 | Model 2 | Model 3 | Model 4 | Model 5** |

| Sex (females = 0, males = 1) | -0.143* | -0.131* | -0.139* | -0.135* | -0.135* |
| --- | --- | --- | --- | --- | --- |
| Age (years) | 0.270* | 0.270* | 0.251* | 0.249* | 0.253 |
| BMI (kg/m^2^) |  | -0.152* | -0.141* | -0.140* | -0.140* |
| Vitamin D intake (ug/d)§ |  |  | 0.274* | 0.287* | 0.287* |
| Calcium intake (mg/d)§ |  |  |  | -0.047* | -0.045* |
|  |  |  |  |  |  |
| R^2^ | 0.092 | 0.115 | 0.189 | 0.191 | 0.220 |

*P < 0.001. **Adjusted for month of blood sampling using dummy variables

§Including supplements
